# A Case of Corneal Thinning During S-1 Oral Administration

**DOI:** 10.7759/cureus.58356

**Published:** 2024-04-15

**Authors:** Ayaka Shimada, Mizuki Iida, Kana Murakami, Sho Ichioka, Akiko Harano, Masaki Tanito

**Affiliations:** 1 Department of Ophthalmology, Faculty of Medicine, Shimane University, Izumo, JPN

**Keywords:** corneal thinning, corneal astigmatism, decreased vision, ocular side effects, anticancer drug, cornea

## Abstract

We experienced a case of bilateral corneal thinning during the oral taking of S-1, a combination anti-cancer drug of tegafur, gimeracil, and oteracil-potassium. A 69-year-old man was prescribed oral S-1 for the treatment of duodenal papilla adenocarcinoma and intraductal papillary mucinous neoplasm. However, he developed a decrease in visual acuity in both eyes after three cycles of S-1 oral taking, and ophthalmic examination revealed corneal thinning exceeding 100 µm and an increase in high-order irregularity of cornea in both eyes. After one month after discontinuation of S-1, his visual acuity and corneal thickness returned to its previous levels. Besides corneal ulcers and perforations, corneal thinning can be recognized as a potential corneal side effect necessitating monitoring during S-1 treatment.

## Introduction

S-1 is a combination of tegafur, a prodrug of 5-fluorouracil (5-FU), and gimeracil and oteracil-potassium, antimetabolites of 5-FU. 5-FU exhibits an anti-cancer effect by inhibiting thymidylate synthase, leading to the disruption of DNA and RNA synthesis. In Japan, S-1 is approved for various cancers, including gastric, colorectal, and non-small cell lung cancers, among others. Associated with S-1, ocular side effects such as corneal abrasions, lacrimal duct stenosis, and conjunctivitis have been reported [1]. We experienced a case of corneal thinning during S-1 oral taking.

## Case presentation

The 69-year-old man had been treated for open-angle glaucoma in both eyes (OU) for four years. His eye medical history included cataract surgery in the right eye (OD) and both small-incisional cataract extraction with intraocular lens (IOL) implantation and microhook trabeculotomy in the left eye (OS). For the past 10 months, he has been using two types of eye drop medications: timolol maleate (tafluprost) and brimonidine tartrate (brinzolamide) OU.

He visited our department every three months for regular follow-ups. During his last six visits, the average central corneal thickness was measured at 504.7 μm OD and 506.3 μm OS using the EM-3000 specular microscope (Tomey, Nagoya, Japan). Following these visits, the patient underwent pancreaticoduodenectomy for duodenal papilla adenocarcinoma and intraductal papillary mucinous neoplasm. One week after the surgery, while still hospitalized, his visit to our department revealed a best-corrected visual acuity (BCVA) of 1.2 OU with a refractive correction of -3.0 diopters (D). The intraocular pressures (IOPs) were 13 mmHg OD and 14 mmHg OS. Using the specular microscope again, the central corneal thickness was measured at 492 μm OD and 498 μm OS.

**Figure 1:**
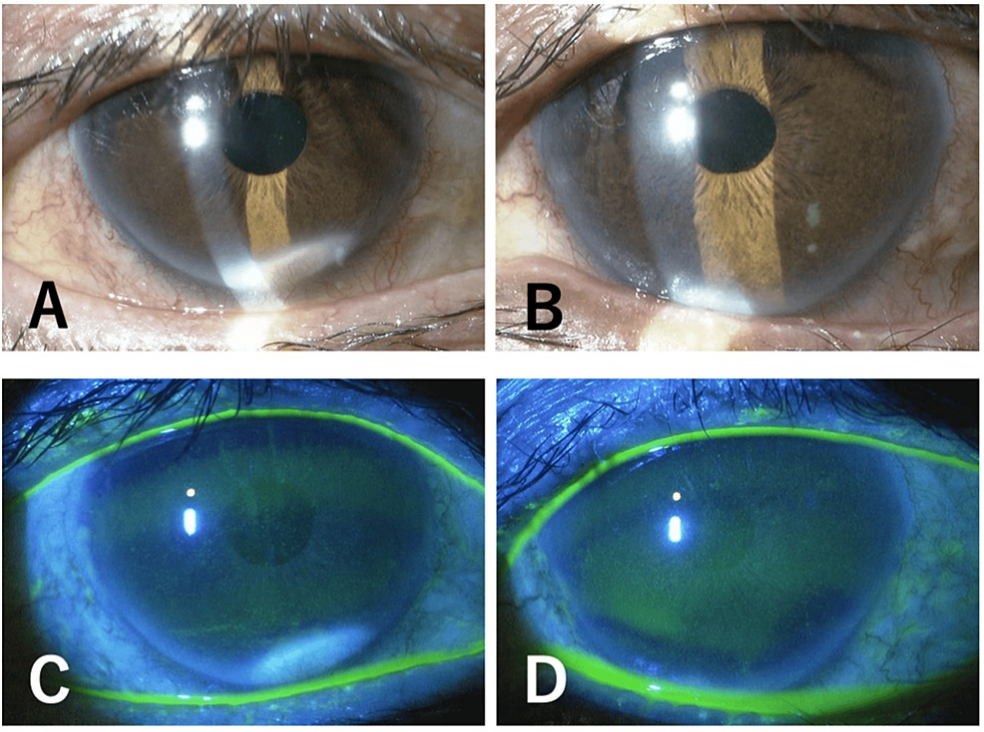
Slit-lamp findings (A, B) and fluorescein staining (C, D) in the right (A, C) and left (B, D) eyes one month after starting S-1.

**Figure 2:**
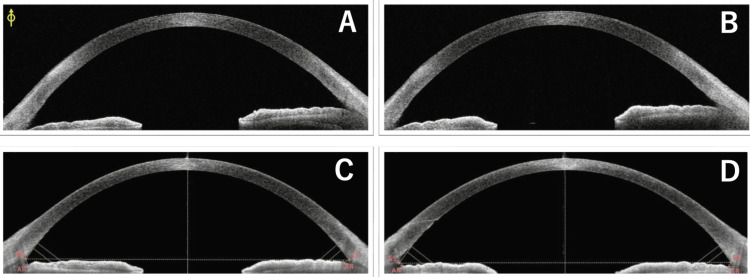
Corneal scans by anterior-segment OCT in the right (A, C) and left (B, D) eyes before (A, B) and one month after (C, D) starting S-1.

**Figure 3:**
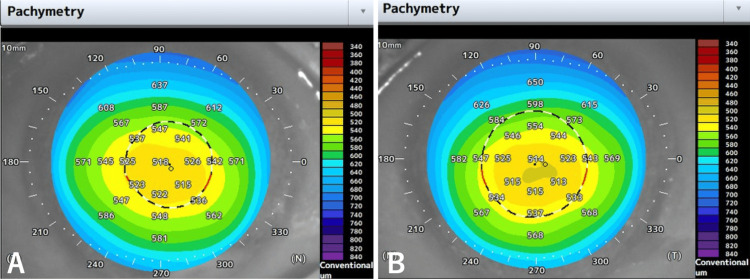
Corneal pachymetry in the right (A) and left (B) eyes before starting S-1.

**Figure 4:**
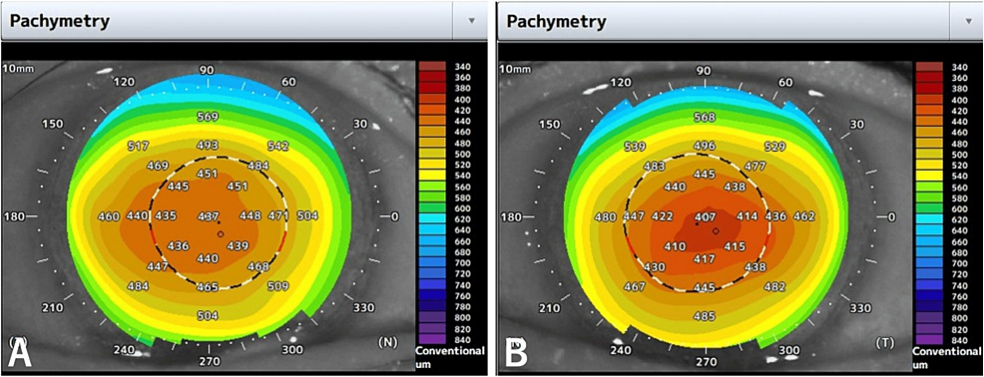
Corneal pachymetry in the right (A) and left (B) eyes one month after starting S-1.

**Figure 5:**
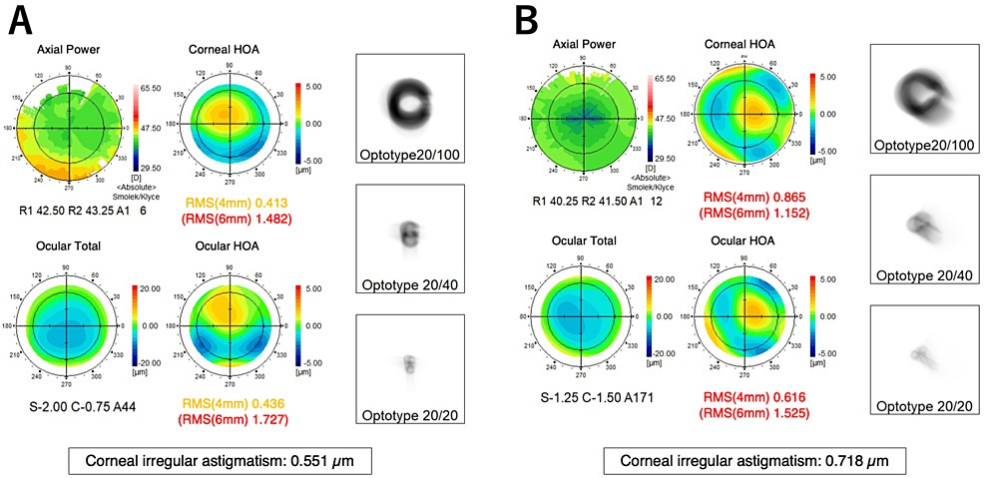
Wavefront analysis results of the right (A) and left (B) eyes one month after starting S-1.

One month after the surgery, he commenced chemotherapy with oral S-1, undergoing cycles of 50 mg daily for two weeks, followed by a one-week interval. No other anticancer drugs were used during this period. Upon completing three cycles, he experienced blurred vision OU and independently decided to discontinue S-1 midway through the fourth cycle. He returned to our department after two weeks of persistent blurred vision. At that time, his BCVA was 0.4 OD and 0.2 OS, with a refractive correction of -2.5 D for both eyes. His IOPs were measured at 11 mmHg OU. Slit-lamp examination revealed superficial punctate keratopathy in both eyes; no significant new corneal opacity was noted, except for pre-existing peripheral corneal opacity in both eyes (Figure 1). No opacity was observed in the IOL or vitreous cavity. Fundus examination and retinal optical coherence tomography (OCT) (RS-3000 Advance 2; Nidek, Gamagori, Japan) showed no changes from the previous visit. Tomographic analysis using anterior-segment (AS) OCT (Casia 2 Advance; Tomey Corporation, Nagoya, Japan) revealed remarkable corneal thinning compared to before S-1 treatment in both eyes (Figure 2). Pachymetric analysis of the AS-OCT images showed central corneal thicknesses of 518 μm OD and 514 μm OS before S-1 taking (Figure 3) became 436 μm OD and 407 μm OS at this visit (Figure 4). One week later, his BCVA improved to 0.9 OD and 0.4 OS. Wavefront analysis (WFA) using a wavefront analyzer (KR-1W; TOPCON Healthcare, Tokyo, Japan) calculated the corneal irregular astigmatism to be 0.551 μm OD and 0.718 μm OS (Figure 5).

**Figure 6:**
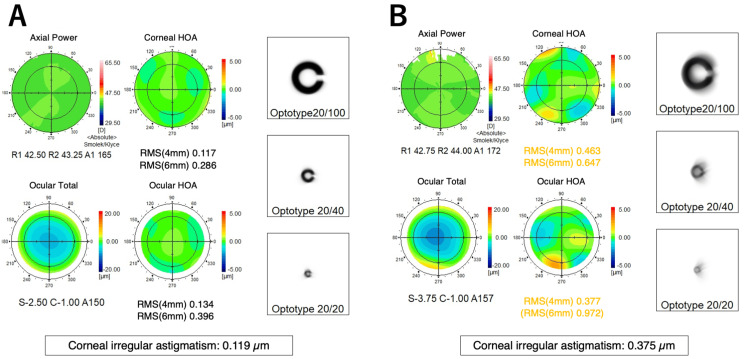
Wavefront analysis results of the right (A) and left (B) eyes one month after discontinuing S-1.

One month after discontinuing S-1, his BCVA improved to 1.0 OU. Central corneal thickness, measured by AS-OCT, increased to 525 μm OD and 511 μm OS. Corneal irregular astigmatism, assessed by WFA, decreased to 0.119 μm OD and 0.375 μm OS (Figure 6). He resumed S-1 treatment the same day and was advised to use artificial tear eyedrops (Soft Santear; Santen Pharmaceuticals, Osaka, Japan) many times daily to rinse out any S-1 residues in tears, suspecting that S-1 might have induced the corneal thinning. After completing three more cycles of S-1 treatment, his BCVAs remained stable at 1.2 OU. However, the central corneal thickness slightly decreased to 508 μm OD and 498 μm OS. Corneal irregular astigmatism by WFA was measured at 0.409 μm OD and 0.373 μm OS.

## Discussion

We encountered a case of bilateral corneal thinning. The patient experienced blurred vision after three cycles of S-1 treatment, which was accompanied by corneal thinning and higher-order corneal aberrations. We noted that the corneal thickness returned to its original level one month after discontinuing S-1, prompting us to investigate the possible correlation between S-1 and these ocular changes.

S-1 is known to cause ocular side effects; approximately 8% of patients experience lacrimal duct disorders and 6% develop corneal epithelial disorders following oral administration of S-1 [2]. The presence of 5-FU in the tear fluid of patients undergoing S-1 treatment is believed to be a contributing factor to these ocular side effects [3]. There have been two reported instances of corneal perforation associated with the use of S-1. In one case, the condition progressed to perforation following recurrent corneal erosions [4]. Our case, featuring reversible corneal thinning without corneal erosion, is unique in the existing literature.

Our case demonstrated a reduction in corneal thickness exceeding 100 µm in both eyes (OU), constituting a 20% decrease in total corneal thickness. Given that approximately 90% of a healthy cornea's thickness comprises stromal tissue, the reduction observed in our case primarily affected the stromal layer. Confocal microscopy of corneal abrasions in 20 patients receiving oral S-1 administration revealed infiltration of inflammatory cells into the epithelial basement membrane and the superficial stromal layers in 85% of the cases [5]. This observation underscores the significant potential impact of S-1 oral administration on corneal stromal tissue.

The precise mechanism behind the reversible change in corneal thickness caused by S-1 remains unclear. Apart from S-1, there have been reports of corneal thinning and the development of subacute cataracts linked to FGFR inhibitors. With the exception of one case involving ulcer formation, all reported cases showed a reduction in visual acuity attributed to superficial punctate keratitis and corneal thinning (440-470 µm) within three to six months after initiating treatment with erdafitinib. An improvement was noted following the discontinuation of erdafitinib and the application of dry eye drops. Both FGFR inhibitors and 5-FU impact cell division, suggesting a possible connection between their effects and the healing process of corneal epithelium and stroma, which may lead to corneal thinning [6].

## Conclusions

We encountered a case of corneal thinning subsequent to starting oral administration of S-1, presumably affecting the stromal layer. Inflammation within the corneal stroma, attributed to 5-FU present in tear fluid, has been linked to corneal thinning. Besides corneal ulcers and perforations, corneal thinning can be recognized as a potential ocular side effect necessitating monitoring during S-1 treatment.
